# Oxygen Concentration and Oxidative Stress Modulate the Influence of Alzheimer's Disease A*β*_1–42_ Peptide on Human Cells

**DOI:** 10.1155/2018/7567959

**Published:** 2018-01-11

**Authors:** Tamara Džinić, Norbert A. Dencher

**Affiliations:** ^1^Physical Biochemistry, Department of Chemistry, Technische Universität Darmstadt, D-64287 Darmstadt, Germany; ^2^Research Center for Molecular Mechanisms of Aging and Age-Related Neurodegenerative Diseases, Moscow Institute of Physics and Technology, Dolgoprudny, Russia

## Abstract

Reactive oxygen species (ROS) generated after exposure to ionizing radiation and toxic peptides, in mitochondrial metabolism and during aging contribute to damage of cell's structural and functional components and can lead to diseases. Monomers and small oligomers of amyloid beta (A*β*) peptide, players in Alzheimer's disease, are recently suggested to be involved in damaging of neurons, instead of extracellular A*β* plaques. We demonstrate that externally applied disaggregated A*β*_1–42_ peptide interacts preferentially with acidic compartments (lysosomes). We compared standard cell cultivation (21% O_2_) to more physiological cell cultivation (5% O_2_). Cells did not exhibit a dramatic increase in ROS and change in glutathione level upon 4 *μ*M A*β* peptide treatment, whereas exposure to 2 Gy X-rays increased ROS and changed glutathione level and ATP concentration. The occurrence of the 4977 bp deletion in mtDNA and significant protein carbonylation were specific effects of IR and more pronounced at 21% O_2_. An increase in cell death after A*β* peptide treatment or irradiation was unexpectedly restored to the control level or below when both were combined, particularly at 5% O_2_. Therefore, A*β* peptide at low concentration can trigger neuroprotective mechanisms in cells exposed to radiation. Oxygen concentration is an important modulator of cellular responses to stress.

## 1. Introduction

Oxidative damage caused by ROS generated either as by-products of cell metabolism and radiation or during aging alters the vitality of cells and contributes to diseases such as cancer, neurodegeneration, and cardiovascular diseases. Persistent and long-term action of ROS in cells can result in a permanent damage despite the low ROS production under physiological conditions [1]. Proteins are one of the major targets of oxidative stress, which also can have detrimental effects on other cellular components (i.e., nucleic acids and lipids). For example, the mitochondrial genome is in close proximity to the ROS production site in the mitochondria (i.e., the respiratory chain) and is less protected by stabilizing proteins and therefore is highly susceptible to oxidative damage which accumulates with aging of, for example, the human brain [2] and leads to alterations expressed in Alzheimer's disease (AD) too [3, 4]. Age is a major risk for pronounced oxidative damage of the organism as well as for AD. The disease was described more than hundred years ago [5] and is still incurable due to its complexity and lack of understanding of its cause(s) despite modern technology and tremendous scientific efforts. Although a growing amount of evidence has pointed out the inconsistency of the amyloid cascade hypothesis [6] as reviewed by Herrup [7], amyloid beta (A*β*) peptide is still discussed as an important (but not the sole) player in Alzheimer's disease [8, 9]. Although fibrillar A*β* peptides aggregate in the form of extracellular plaques in the brain and represent a clinical hallmark of AD, A*β* peptide was found within neurons of AD human brains as well [10]. A*β* oligomers are toxic forms of the peptide as reviewed by Stefani [11]. A*β* monomers and small oligomers interact with model lipid membranes, by deep penetration into the membrane [12, 13] and by induction of channels [14]. Mitochondria of SH-SY5Y cells as well as those of neurons of human brain import A*β* peptide through TOM (translocase of the outer membrane) complex [15]. Mitochondrial dysfunction as a result of mtDNA damage, changes in the number of oxidative phosphorylation subunits, and abnormalities of fission and fusion processes of the organelle as well as disruption of protein maturation and import into mitochondria are discussed as early events in AD [16–18]. Very high level of oxidative stress affects A*β* peptide trafficking with the increase of intralysosomal A*β* content through activation of macroautophagy [19]. Furthermore, amylospheroids (ASPD) containing A*β* peptide oligomers interact with the *α*-subunit of neuron-specific Na^+^/K^+^-ATPase (NAK*α*3) resulting in presynaptic calcium overload and neuronal death [20]. However, exact mechanisms of A*β* peptide-induced alterations are still obscure. On the list of other possible causative agents and factors for the development of AD is ionizing radiation (IR), particularly dental X-rays and related IR capable of destroying dividing microglial cells that support neurons [21], by damaging microglia telomeres causing premature death as proposed by Rodgers [22]. Furthermore, mitochondria are very important targets of ionizing radiation [23] and their direct damage leads to further nuclear DNA damage [24]. Accumulation of a common deletion in mtDNA (Δ-mtDNA^4977^) occurs after mitochondrial degeneration in diseases and aging and is induced by ionizing radiation as well [25, 26].

Since oxygen in the cell culture modulates cellular response to stress [23], we studied effects of ionizing radiation or A*β*_1–42_ peptide disaggregated to monomers and oligomers on human cells pretreated with retinoic acid for induction of differentiation at two different oxygen concentrations (21% and 5% O_2_). For the first time, to our knowledge, the combined effects of the A*β* peptide and ionizing radiation on cellular parameters and survival were investigated. We observed the accumulation of A*β*_1–42_ preferentially in acidic organelles (lysosomes and likely late endosomes) of SH-SY5Y cells cultivated at both 21% and 5% O_2_ and only to a minor extent in other organelles (mitochondria and endoplasmatic reticulum). A*β*_1–42_ peptide or 2 Gy X-ray alone results in an increase in cell death. Interestingly, the combination of A*β* peptide treatment and irradiation led to decreased level of cell death even below the level of death in control cells, particularly at 5% O_2_. Our data reveal complex interplay of ionizing radiation and amyloid beta peptide depending on the oxygen concentration in the cell culture which modulates cellular responses to stress.

## 2. Materials and Methods

### 2.1. Cell Culture Conditions and Treatments

Human neuroblastoma (SH-SY5Y) cells were grown in DMEM (Gibco, Life Technologies, Paisley, UK) supplemented with 10% FBS (PAA Laboratories GmbH, Pasching, Austria), 2% L-glutamine, and 5 U/ml penicillin/5 *μ*g/ml streptomycin (Gibco, Life Technologies, Paisley, UK) at 37°C, 5% CO_2_, at two different oxygen conditions (standard 21% O_2_ and 5% O_2_, resp.) as described previously [23]. Simple home-made incubators for cultivation of cells at 5% O_2_ were set up according to the protocol by Wright and Shay [27]. Cells were first cultivated (including regular passaging to obtain enough cells) at 5% O_2_ for one week before conducting experiments to ensure enough time for their adaptation to the new oxygen condition and were further maintained at 5% O_2_. Cells at 5% O_2_ were always cultivated and passaged parallel to cells at 21% O_2_ starting with the same number of cells. Cell culture was regularly checked for the presence of mycoplasma using a Mycoplasma Detection Kit (Bimake, Houston, TX, USA). Differentiation of SH-SY5Y cells cultivated under both oxygen conditions was induced by an incubation of ~1 × 10^4^ cells/ml with 10 *μ*M all-*trans* retinoic acid (Sigma-Aldrich, Taufkirchen, Germany) added to the cell culture medium on passage day 0 [28]. After 3 days, cells were harvested for assays using 0.05% trypsin-EDTA (Gibco, Life Technologies, Paisley, UK). Cells were treated with 4 *μ*M A*β*_1–42_ peptide (China Peptides, Shangai, China) or 200 nM FITC-labelled A*β*_1–42_ peptide (Bachem, Bubendorf, Switzerland) disaggregated according to the modified protocol by Jao and colleagues [29]. Briefly, 1 mg amyloid beta peptide was disaggregated in a glass vial using 1.5 ml distilled trifluoroacetic acid (TFA) (Carl Roth GmbH, Karlsruhe, Germany) in an ultrasonic bath (Sonorex TK 52 H, Bandelin electronic GmbH, Berlin, Germany) for 15 min at RT, centrifuged at 3000 ×g, 15 min, and 16°C. The supernatant was transferred into a new glass vial, and N_2_ was used to completely remove TFA. The peptide was dissolved in DMSO (1 mM stock solution for unlabelled and 200 *μ*M for FITC-A*β* peptide) and stored at −20°C in aliquots to avoid repeated freeze-thaw cycles. 6 h after adding A*β*_1–42_ peptide, cells were irradiated once using an Isovolt DS1 X-ray tube (Seifert, Fairview Village, PA, USA) with wolfram anode set to 90 kV, 19 mA, and 30 cm distance from a sample for 40 sec to obtain a dose of 2 Gy. Longer wavelengths (above 0.2 nm) were excluded using a 2 mm aluminum filter. Cells were incubated for the next 18 h or 54 h post irradiation (24 h or 72 h after adding A*β*_1–42_ peptide) (if not otherwise indicated) in appropriate cell culture conditions before conducting experiments.

### 2.2. Interaction of A*β*_1–42_ with SH-SY5Y Cells

#### 2.2.1. Flow Cytometry

Cells were incubated with disaggregated 200 nM FITC-labelled A*β*_1–42_ peptide (Bachem, Bubendorf, Switzerland): 5, 10, 15, and 30 min and 1, 3, 18, and 24 h. Cells were trypsinized, washed, and resuspended in PBS (Gibco, Life Technologies, Paisley, UK), and the fluorescence signal was measured by flow cytometry (S3 Cell Sorter (Bio-Rad Laboratories, Hercules, CA)) using 488 nm laser excitation. 5000 cells were analyzed, and fluorescence signals were plotted in Kaluza software (version 1.3) (Beckman Coulter Inc., Indianapolis, IN, USA) as FL1-area log against the signal count for the detection of the shift of fluorescence signal compared to the unstained cells. Cells used in this experiment were nondifferentiated. For all other experiments, differentiation of cells was induced using retinoic acid.

#### 2.2.2. Confocal Microscopy

About 5 × 10^4^ cells/ml were grown on 25 mm round glass coverslips (Carl Roth GmbH, Karlsruhe, Germany) and incubated for 3, 8, and 18 h with 400 nM FITC-labelled A*β*_1–42_ peptide. Imaging was performed in PBS containing 5% FBS at room temperature using the Leica confocal system TCS SP5 II with the software LAS AF (version 2.60) (Leica Microsystems CMS GmbH, Heidelberg, Germany). Incubation with ER-Tracker™ Red (*λ*_EX_/*λ*_EM_ = 587/615 nm), MitoTracker® Red CM-H_2_Xros (*λ*_EX_/*λ*_EM_ = 579/599 nm), or LysoTracker® Red (*λ*_EX_/*λ*_EM_ = 577/590 nm) (Molecular Probes, Invitrogen, Eugene, OR) was performed 15 or 5 min (for LysoTracker Red) prior to imaging. FITC and ER-Tracker/MitoTracker/LysoTracker Red were sequentially excited with an argon laser at 488 nm and with a yellow diode at 561 nm, respectively. Images (512 × 512 pixels) were acquired by sequential scanning between lines (line average 6) using 40x (1.3 NA) oil-immersion objective with a 12-bit HyD detector at corresponding spectral range for each fluorophore. Images were overlayed in ImageJ software (version 1.48) (http://imagej.nih.gov/ij/) for putative detection of fluorescence signal colocalization.

### 2.3. ROS Level

Cells were seeded to 10 cm^2^ Petri dishes at a density of ~1 × 10^5^ cells/ml. On the day of the assay, cells were harvested by trypsinization and incubated in 1 ml PBS containing 5% FBS with 20 *μ*M carboxy-H_2_DCFH-DA (2′7′-dichlorofluorescin diacetate (C-DCF)) (Molecular Probes Invitrogen, Eugene, OR, USA) for 20 min at 37°C. Following incubation, cells were pelleted (700 ×g, 5 min, RT) and resuspended in 0.5 ml PBS. Cells treated with 1 mM H_2_O_2_ in PBS containing 5% FBS for 30 min at RT were used as a positive control for an increase in ROS. Fluorescence intensity of C-DCF (*λ*_EX_/*λ*_EM_ = 492–495/517–527 nm) was measured by flow cytometry using the S3 Cell Sorter. Data were analyzed by Kaluza software as FL1-area log against the signal count for the detection of the shift of fluorescence signal compared to the positive control (H_2_O_2_-treated cells).

### 2.4. Glutathione Level

The level of glutathione was measured using an EarlyTox Glutathione Assay Kit (Molecular Devices, Sunnyvale, CA, USA). ~2 × 10^4^ cells were seeded per well (100 *μ*l culture medium) of a 96-well black clear F-bottom plate (Greiner bio-one GmbH, Frickenhausen, Germany) and incubated at 37°C and at 21% and 5% O_2_, respectively, overnight. Cells treated with 2 *μ*M staurosporine (Cell Signaling Technology, Danvers, MA, USA) which inhibits protein C kinase and other kinases leading to cell death, and a decrease in GSH served as a positive control for a decrease in GSH level. The assay was performed 1 h and 18 h after X-ray irradiation or 6 h and 24 h after A*β* peptide addition by adding 40 *μ*M monochlorbimane (MCB) directly to the cell culture media. Cells were incubated at 37°C, and fluorescence of the MCB-S-glutathione conjugate was measured using an Infinite M1000 plate reader (Tecan Group Ltd., Männedorf, Switzerland) with the 394 nm excitation filter and 490 nm emission filter. The intensity of the fluorescence signal is directly proportional to the level of GSH in the cells.

### 2.5. Cellular ATP Concentration

Total cellular ATP concentration of SH-SY5Y cells was determined using a luminescent ATP detection assay (ab113849, Abcam, Cambridge, UK) according to the manufacturer's protocol and as described previously [23].

### 2.6. Oxyblot

In order to determine the overall degree of oxidation (carbonylation) of total cellular proteins, Oxyblot assay was performed using an OxyBlot™ Protein Oxidation Detection Kit (Merck Millipore, Billerica, MA, USA) according to the manufacturer's protocol with modifications. Cells were lysed using RIPA buffer (50 mM Tris-HCl (pH 7.4), 1% (*v*/*v*) IGEPAL®-CA630 detergent (Sigma-Aldrich, Saint Louis, MO, USA), 0.5% Na-deoxycholate, 0.1% SDS, 150 mM NaCl, 2 mM EDTA, and 50 mM NaF) with protease inhibitor cocktail (Sigma-Aldrich, Taufkirchen, Germany) in 1 : 200 ratio using 24 *μ*l buffer per ~2 × 10^5^ cells on ice. Samples were centrifuged at 14000 ×g for 15 min at 4°C, and supernatants were stored at −20°C. Protein concentrations were determined by Bradford assay using Roti®-Nanoquant (Carl Roth GmbH, Karlsruhe, Germany). Proteins from cell lysates were assessed using anti-DNP (dinitrophenylhydrazone) antibody for carbonyl groups introduced into protein side chains by oxidative reactions with reactive oxygen species. Oxidized BSA served as a positive control for protein oxidation and was prepared as follows: 10 mg/ml BSA was incubated for 5 h at 37°C in a buffer for positive control (25 mM HEPES, 25 mM ascorbic acid (Na-salt), and 100 *μ*M FeCl_3_, pH 7.2) and dialyzed overnight (12–16 kDa cut-off membrane) in a dialysis buffer (50 mM HEPES, 1 mM EDTA). Each sample was denatured by adding 12% SDS to a final concentration of 6% SDS. Samples were derivatized by adding 10 *μ*l of 1x 2,4-dinitrophenylhydrazine (DNPH Solution from the kit) except for the negative control for immunobinding (BSA, prepared as described above, to which 10 *μ*l 1x derivatization-control solution was added instead of DNPH solution). About 15 *μ*g protein from cell lysates was loaded per lane of a 9% SDS gel. Following electrophoresis, the nitrocellulose membrane was immunoblotted on primary 2,4-dinitrophenylhydrazone (DNP) rabbit antibody (1 : 500) from the kit and secondary donkey anti-rabbit IgG HRP antibody (dilution 1 : 2000) (Santa Cruz Biotechnology Inc., Dallas, TX, USA). ECL-based detection of signal on nitrocellulose membrane with luminol was performed using the CCD camera system Fujibox with Image Reader LAS-3000 software (Fujifilm Holdings K.K., Tokyo, Japan). Quantification of the oxidized proteins was performed with ImageJ software (http://imagej.nih.gov/ij/), and data is expressed as a ratio of oxyblot lane average intensity (mean gray value) and control band intensity.

### 2.7. mtDNA Amount

Total genomic DNA was isolated using the Blood & Cell Culture DNA Mini Kit (Qiagen, Hilden, Germany) according to the manufacturer's protocol. DNA was quantified using PicoGreen dye (*λ*_EX_/*λ*_EM_ = 480/520 nm) (Molecular Probes, Eugene, OR, USA) that binds to dsDNA. Lambda/HindIII DNA Digest (New England Biolabs, Ipswich, MA, USA) was used for generating a standard curve (1.25–10 ng/*μ*l DNA) for determining concentrations of samples. Fluorescence of the dye was measured using an Infinite M1000 plate reader with a 485 nm excitation filter and 535 nm emission filter. To determine mitochondrial DNA amount, short fragments of the mtDNA were amplified since there is a low probability of damaged DNA in such fragments [30]. In the wild-type mtDNA, forward (5′-CTGAGCCTTTTACCACTCCAG3′) and reverse (5′-GGTGATTGATACTCCTGATGCG-3′) primers (http://www.biomers.net) located within the deletion region (to ensure that the mtDNA with a deletion of 4977 bp is not amplified) yield a PCR product of 142 bp [31]. Standard PCR reaction for the determination of mitochondrial DNA amount was set up as follows: 2x Taq Master Mix (New England Biolabs, Ipswich, MA, USA), 10 *μ*M forward and 10 *μ*M reverse primer, 200 ng template DNA, and was performed in a total volume of 50 *μ*l and at appropriate cycling conditions (94°C for 5 min; 35 cycles at 94°C for 20 s, 60°C for 20 s, and 72°C for 20 s; a final extension at 72°C for 2 min) in MyCycler™ Thermal Cycler (Bio-Rad Laboratories, Hercules, CA). The PCR products were loaded on 2% agarose (peqlab, Erlangen, Germany) gels with 0.25 *μ*g/ml ethidium bromide (Carl Roth GmbH, Karlsruhe, Germany) for visualization. Products were quantified using PicoGreen dye. Relative fluorescence values were obtained by subtracting the fluorescence value of the negative control (PCR assembly without DNA) from each sample.

### 2.8. Detection of Common mtDNA Deletion (Δ-mtDNA^4977^)

Nested PCR was used for detecting common mtDNA deletion (Δ-mtDNA^4977^). In a first PCR step, a region outside the 13 bp repeats, where the deletion occurs, was amplified using primers: AACCACAGTTTCATGCCCATC (forward) and TGTTAGTAAGGGTGGGGAAGC (reverse). In a second PCR step, 1 *μ*l of this primary reaction was used as a template for the amplification of the aberrant mtDNA using primers: ACCCTATTGCACCCCCTCTAC (forward) and CTTGTCAGGGAGGTAGCGATG (reverse) [32]. Reaction setup and cycling conditions for primary and secondary reactions were similar: predenaturation at 94°C for 5 min; 35 cycles at 94°C for 20 s, 58°C for 45 s (or 60°C for 40 sec), and 72°C for 50 s (or 45 sec); and a final extension at 72°C for 5 min. The presence of the deletion was detected in the form of a 358 bp band on 2% agarose gel. Products were quantified using PicoGreen dye as described above.

### 2.9. Cell Death Detection

In order to determine the percentage of apoptotic and necrotic cells upon X-ray irradiation, treatment with A*β*_1–42_, and the combination of both, cells were analyzed for Annexin V-FITC staining and propidium iodide (PI) staining. Cells (seeded at a density of ~1 × 10^5^ cells/ml) were harvested and pelleted by centrifugation (700 ×g for 5 min at 4°C). Cells were resuspended in 300 *μ*l 1x Binding Buffer (Biotool, Houston, TX, USA) and incubated with 0.5 *μ*g (10 *μ*l) propidium iodide (Molecular Probes, Eugene, OR, USA) and 0.4 *μ*g (2 *μ*l) Annexin V-FITC (Biotool, Houston, TX, USA) for 15 min at room temperature in the dark. Treatment of cells with 2 *μ*M staurosporine for 24 h was used as a positive control for apoptosis and incubation of cells at 60°C for 15 min served as a control for necrosis. 10,000 cells were analyzed by flow cytometry (S3 Cell Sorter). Fluorescence signal of annexin V-FITC was measured using 488 nm laser excitation and that of PI using 561 nm laser excitation. Signals were compensated in Kaluza software using single-stained controls for apoptosis and necrosis, respectively.

### 2.10. Statistics

Data were analyzed using two-way ANOVA test with multiple comparison tests (Tukey's or Dunnet's) in GraphPad Prism (version 7) (GraphPad Software Inc., La Jolla, CA, USA) with *p* < 0.05 considered significant (^∗^ *p* < 0.05, ^∗∗^ *p* < 0.01, ^∗∗∗^ *p* < 0.001, and ^∗∗∗∗^ *p* < 0.0001).

## 3. Results

### 3.1. Intracellular Localization of Externally Applied A*β*_1–42_ Peptide

**Figure 1:**
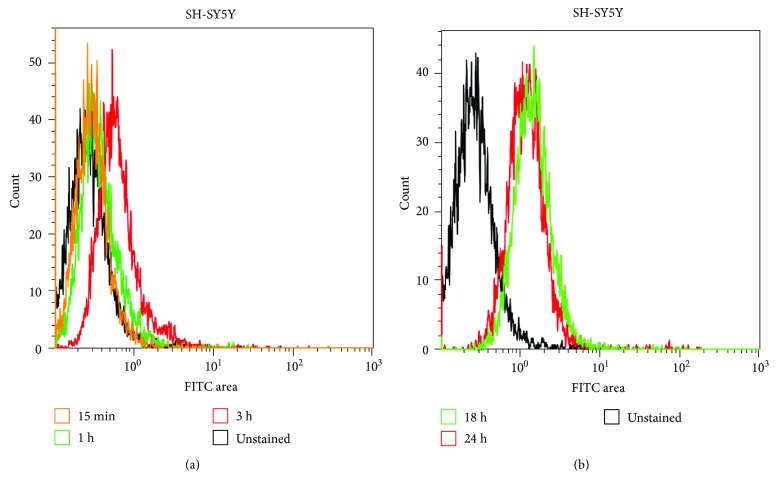
FITC-labelled A*β*_1–42_ peptide interacts with SH-SY5Y cells. Flow cytometry analysis: shift in the fluorescence signal in SH-SY5Y cells cultivated at 21% O_2_ incubated with FITC-A*β*_1–42_ peptide after 15 min, 1, 3, 18, and 24 h compared to unstained cells. Maximum fluorescence signal occurred after 18 h, whereas a slight decrease of the signal was detected after 24 h.

**Figure 2:**
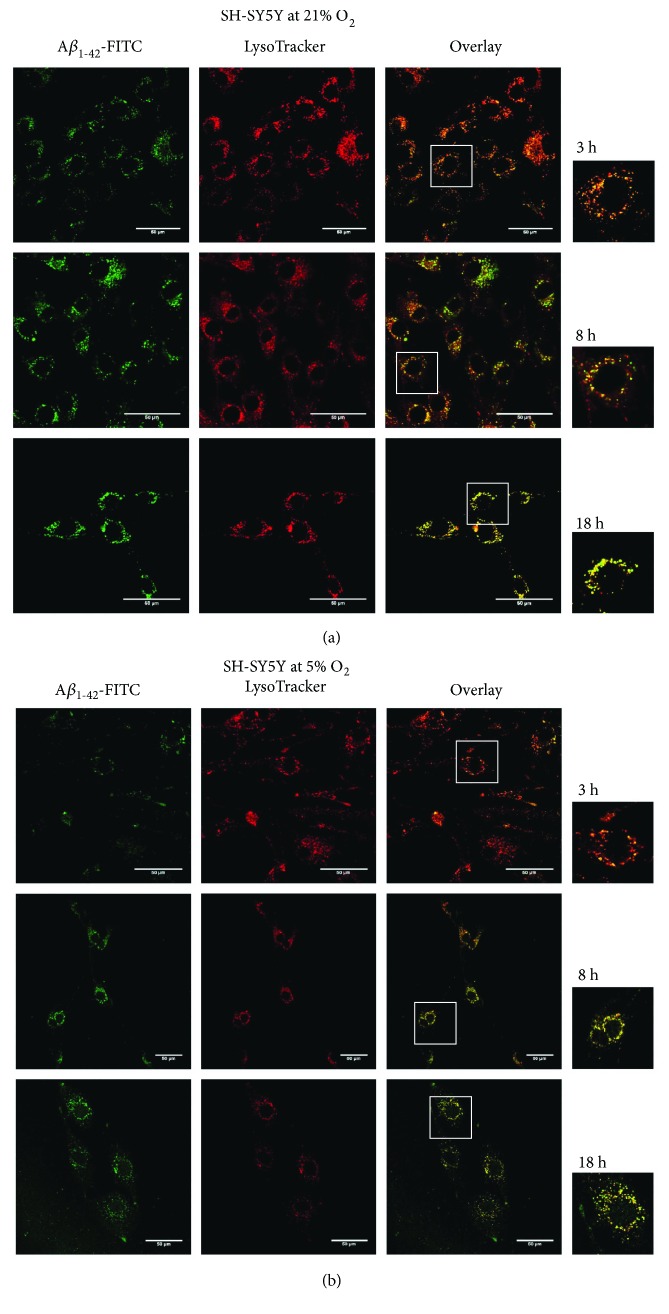
FITC-labelled A*β*_1–42_ peptide interacts with lysosomes of SH-SY5Y cells. FITC-A*β*_1–42_ peptide (green fluorescence) interacts with acidic organelles (lysosomes; late endosomes as dotted structures in selected regions (white squares) of overlay images enlarged on the right side of the panel) stained by LysoTracker Red dye (red fluorescence) at both 21% and 5% O_2_; progressive interaction after 3, 8, and 18 h. 400x magnification. 50 *μ*m scale bar.

**Figure 3:**
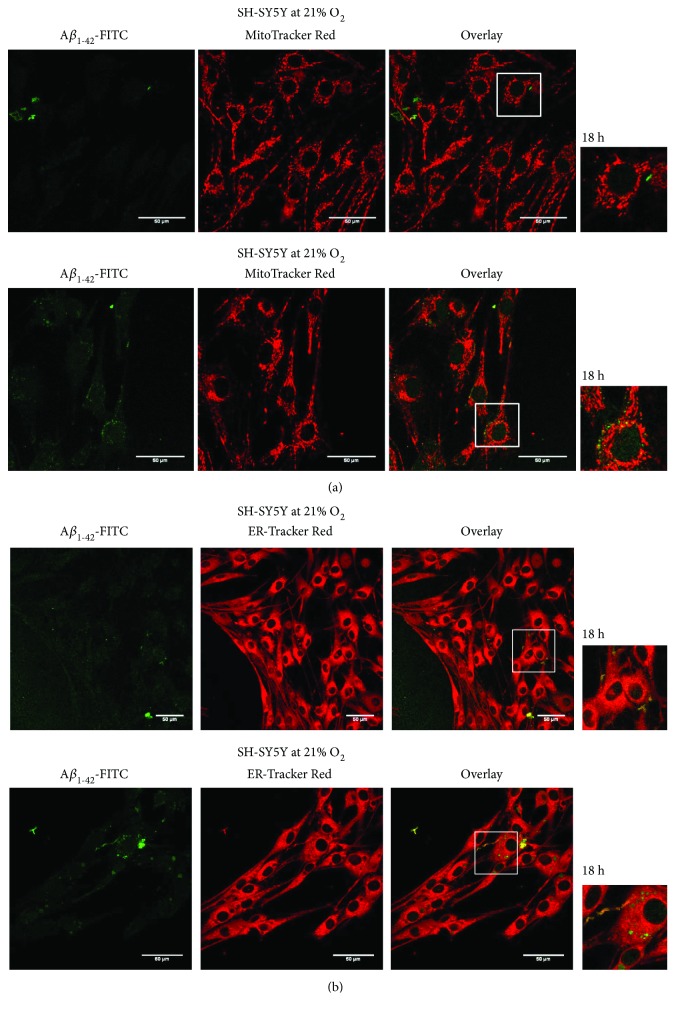
Interaction of FITC-A*β* peptide with the mitochondria and endoplasmatic reticulum. Weak colocalization of FITC-A*β* peptide with the mitochondria stained by MitoTracker Red (red fluorescence) (a) and with endoplasmatic reticulum stained by ER-Tracker Red dye (red fluorescence) (b) at both 21% and 5% O_2_, documented after 18 h. Selected regions (white squares) of overlay images enlarged on the right side of the panel for better visualization. 400x magnification. 50 *μ*m scale bar.

We assayed interaction of the amyloid beta peptide with SH-SY5Y cells using flow cytometry for the detection of the fluorescence signal of FITC-labelled A*β*_1–42_ peptide after 5, 10, 15, and 30 min and after 1, 3, 18, and 24 h. A*β*_1–42_ peptide interacted with SH-SY5Y cells indicated by a progressive increase (shift) in the fluorescence signal after 15 min, 1 h, 3 h, and 18 h of incubation compared to control (unstained) cells (Figure 1). Maximum fluorescence shift was observed after 18 h; thereafter, a slight decrease occurred after 24 h. Figure 1 depicts the data for cells cultivated at 21% O_2_. In order to determine the site(s) and kinetics of interaction, subcellular localization of externally applied A*β*_1–42_ peptide was investigated using confocal microscopy by measuring colocalization of FITC-labelled peptide with organelles (lysosomes, mitochondria, and endoplasmatic reticulum) stained with specific dyes. A*β* peptide strongly interacted with acidic organelles (lysosomes and possibly endosomes) in line with supporting data [33], stained by LysoTracker Red dye, at both 21% and 5% O_2_ with progress in the signal after 3, 8, and 18 h (Figure 2). Only weak colocalization was found with other organelles of the SH-SY5Y cells examined here (endoplasmatic reticulum and mitochondria, resp.) after 18 h at both 21% and 5% O_2_ (Figure 3). Data after 3 and 8 h are displayed in Figure S1.

### 3.2. Changes in Intracellular ROS Level

**Figure 4:**
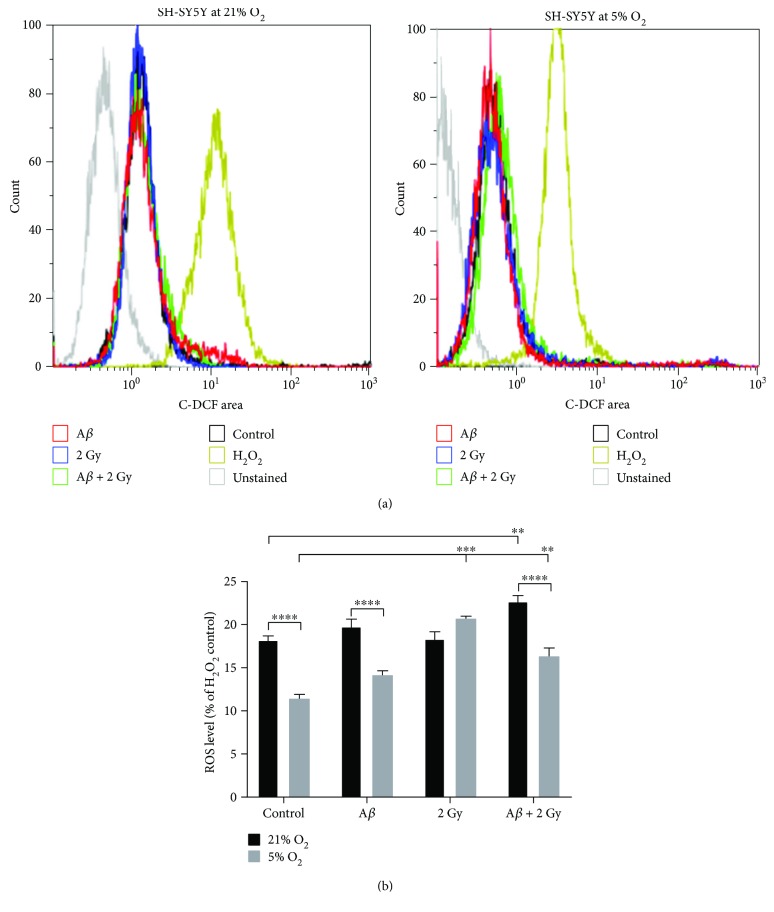
Intracellular ROS level in SH-SY5Y cells. (a) Shift in the fluorescence signal of 2′7′-dihydrofluorescein in comparison to the signal of the positive control (H_2_O_2_-treated cells) and to the nontreated cells detected 18 h after irradiation and 24 h after amyloid beta treatment or combination of both in SH-SY5Y cells depending on the oxygen concentration (21% and 5% O_2_, resp.; results from single measurements by flow cytometry were plotted). (b) Generally higher (~1.5-fold) ROS level was detected in cells cultivated at 21% O_2_ compared to 5% O_2_. A slight (~1.1-fold) increase of ROS level after A*β* peptide treatment at 21% O_2_ and about 1.3-fold at 5% O_2_. A significant (1.8-fold) increase of ROS level after irradiation at 5% O_2_ only and after irradiation of A*β* peptide-treated cells at both 21% (~1.2-fold) and 5% O_2_ (~1.4-fold). Samples were measured at least in duplicates (*n* = 2) in at least three (*N* = 3) independent experiments, and relative fluorescence values are presented as percentages (%) of the fluorescence value of the H_2_O_2_ control (100%). Mean ± SEM analyzed by two-way ANOVA with Tukey's test. (^∗∗^ *p* < 0.01, ^∗∗∗^ *p* < 0.001, and ^∗∗∗∗^ *p* < 0.0001).

Changes of the intracellular ROS level upon irradiation, treatment with A*β*_1–42_ peptide, and the combination of both were monitored by flow cytometry using carboxy-H_2_DCF-DA for the detection of a variety of ROS species such as H_2_O_2_, •OH, and hydroperoxides (Figure 4(a)). Noteworthy, the ROS level in nontreated control cells was significantly higher (~1.5-fold) in cells cultivated at 21% O_2_ (Figure 4(b)). Treatment with A*β* peptide only slightly increased ROS level at both 21% and 5% O_2_ (up to 1.2-fold). 2 Gy X-ray irradiation led to a significantly larger (~1.8-fold) increase in ROS but solely in cells at 5% O_2_. Radiation combined with A*β* peptide treatment resulted in a statistically significant increase in ROS level at both 21% (~1.2-fold) and 5% O_2_ (~1.4-fold) compared to respective controls.

### 3.3. Glutathione Level

**Figure 5:**
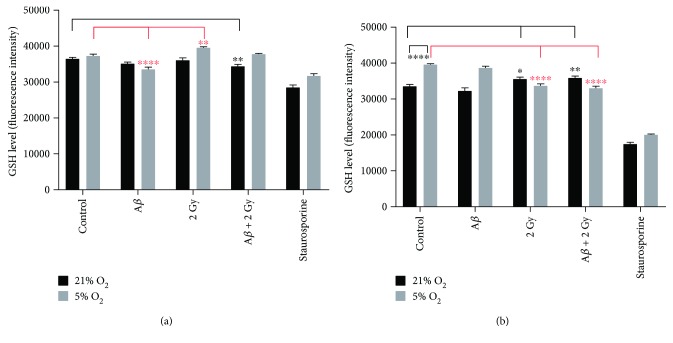
Level of glutathione. SH-SY5Y cells cultivated at 21% and 5% O_2_, respectively, after treatment with A*β* peptide and/or X-ray irradiation compared to nontreated controls. Measurements were performed 6 h after incubation with A*β* peptide and/or 1 h after irradiation (a) and 24 h after incubation with A*β* peptide and/or 18 h after irradiation (2 Gy X-rays) (b), respectively. Samples were measured at least in octuplicates (*n* = 8) in four independent experiments (*N* = 4). Mean ± SEM analyzed by two-way ANOVA with Dunnet's multiple comparison test. (^∗^ *p* < 0.05, ^∗∗^ *p* < 0.01, and ^∗∗∗∗^ *p* < 0.0001).

GSH level is an indicator of cell's antioxidant capacity [34] and was measured in SH-SY5Y cells cultivated at 21% and 5% O_2_ after treatment with A*β* peptide (6 and 24 h, resp.) and/or X-ray irradiation (after 1 h and 18 h, resp.). Varying levels of GSH were detected depending on different durations of A*β* peptide incubation and/or X-ray irradiation (Figure 5). A*β* peptide alone resulted in a statistically significant (~1.2-fold) decrease in GSH level after 6 h of incubation but only in cells cultivated at 5% O_2_ (Figure 5(a)), whereas the small decrease at 21% O_2_ was nonsignificant. 24 h after A*β* peptide treatment, GSH level was at the level of GSH in corresponding nontreated controls at both 21% and 5% O_2_ (Figure 5(b)). 2 Gy X-rays led to an increase in the GSH level only at 5% O_2_ assayed 1 h postirradiation (Figure 5(a)) followed by a decrease 18 h later (Figure 5(b)). On the other hand, at 21% O_2_, irradiation did not lead to a change in GSH level after 1 h, whereas an increase was observed 18 h later. The combination of both A*β* peptide and irradiation resulted in a decrease in the GSH level on the first day and then an increase on the next day in cells at 21% O_2_. On the contrary, this combination of cells at 5% O_2_ did not change GSH level on the first day and then a significant decrease (~1.2-fold) in the GSH level on the next day. Notably, GSH level measured on the second day was significantly (*p* < 0.0001) higher in control cells cultivated at 5% O_2_ compared to that at 21% O_2_. In summary, A*β* peptide by itself did not significantly change the GSH level after 1 day; no matter what the oxygen concentration is in the cell culture. Irradiation alone or in combination with A*β* peptide treatment had opposite effects depending on the oxygen concentration: significantly increased (up to 1.1-fold) GSH level at 21% O_2_ and decreased (up to 1.2-fold) at 5% O_2_, as compared to respective nontreated controls.

### 3.4. Changes in ATP Concentration

**Figure 6:**
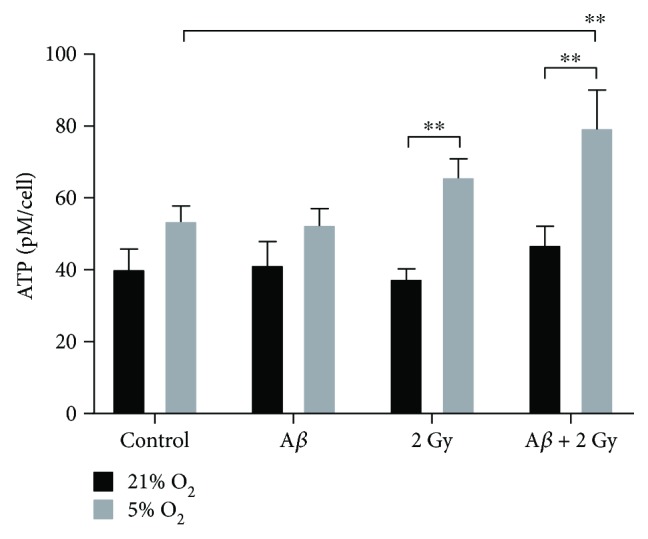
Total cellular ATP concentration. ATP in SH-SY5Y cells cultivated at 21% and 5% O_2_ 24 h after treatment with A*β* peptide and/or 18 h X-ray irradiation, normalized to cell count, and compared to respective controls. ATP concentration was about 1.3- to 1.8-fold higher at all conditions in cells cultivated at 5% O_2_ compared to 21% O_2_. Combination of A*β* peptide treatment and irradiation resulted in a significantly increased (~1.5-fold) ATP concentration at 5% O_2_ compared to the control. Samples were measured at least in duplicates (*n* = 2–4) in three independent experiments (*N* = 3). Mean ± SEM analyzed by two-way ANOVA with Tukey's multiple comparison test with *p* < 0.05 considered as significant. (^∗∗^ *p* < 0.01).

Higher cellular ATP concentration was observed in SH-SY5Y control cells (~1.3-fold) and in all treated cells (1.3- to 1.8-fold) cultivated at 5% O_2_ in comparison to cells at 21% O_2_ (Figure 6). Whereas A*β* peptide did not affect the ATP concentration at these two different oxygen conditions, irradiation alone led to a slight (~1.2-fold) but not a significant increase in ATP and a significant (~1.5-fold) increase when combined with A*β* peptide treatment in cells at 5% O_2_ only.

### 3.5. Protein Carbonylation

**Figure 7:**
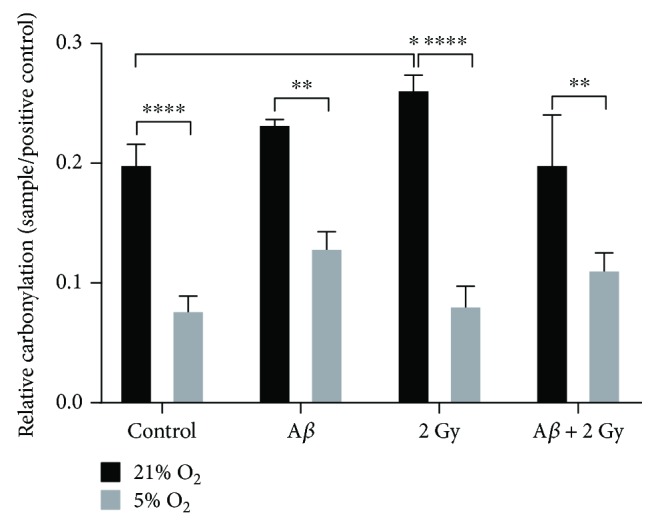
Level of protein carbonylation. Cells cultivated at 21% O_2_ showed severely increased (about 1.8- to 2.5-fold) protein carbonylation compared to cells at 5% O_2_, and this increase at 21% O_2_ only was significantly pronounced 18 h after irradiation. A*β* peptide treatment for 24 h alone or combined with irradiation resulted in an increase (~1.6- and 1.4-fold, resp., not significant) in protein carbonylation in cells at 5% O_2_, whereas only a minor increase (1.15-fold) after A*β* peptide only was observed at 21% O_2_. Combination of two stressors did not lead a further increase in protein carbonylation at 21% O_2_. Samples were measured at least in three independent experiments. Mean ± SEM analyzed by two-way ANOVA with Dunnet's multiple comparison test. (^∗^ *p* < 0.05, ^∗∗^ *p* < 0.001, and ^∗∗∗∗^ *p* < 0.0001).

Intensities of bands in the oxyblot are correlated to the degree of protein carbonylation (assessed using DNP antibody against carbonyl groups) [35, 36]; the more intense the bands are, the higher is the degree of protein oxidation (data in Figure S2). About 1.8- to 2.5-fold higher protein carbonylation was detected in cells cultivated at 21% O_2_ compared to cells at 5% O_2_ at all experimental conditions (Figure 7). Irradiation at 21% O_2_ caused an increase in protein carbonylation, whereas A*β* peptide treatment or the combination of two stressors did not lead to a significant increase in protein carbonylation. An increase (~1.6-fold) in protein carbonylation in cells cultivated at 5% O_2_ was observed after amyloid beta peptide treatment alone and when combined with irradiation (~1.4-fold), whereas irradiation alone did not affect the level of protein carbonylation.

### 3.6. Changes in mtDNA Amount

**Figure 8:**
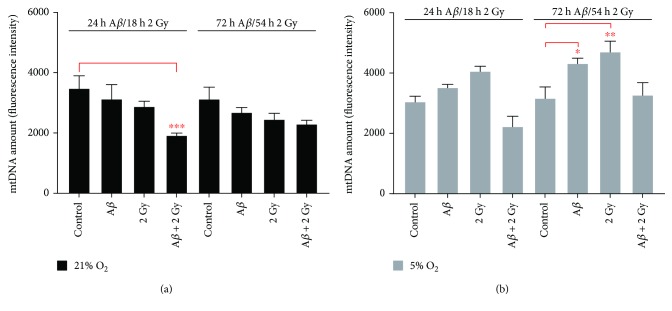
Mitochondrial DNA amount upon A*β*_1–42_ treatment and/or irradiation in SH-SY5Y cells. (a, b) A*β* peptide or irradiation (2 Gy X-rays) alone affected mtDNA amount depending on O_2_ level in the cell culture: about 1.2- and 1.3-fold, respectively, decrease at 21% O_2_ and up to 1.3- and 1.5-fold (a), respectively, increase at 5% O_2_ (b) compared to respective controls. The combined effect of A*β* peptide and irradiation led to a decrease in mtDNA amount at both oxygen concentrations, particularly at 21% O_2_ (1.8-fold) after 1 day/18 h with almost no change observed after 3 days/54 h, whereas at 5% O_2_ it was restored to the level of control sample. Mean ± SEM analyzed by two-way ANOVA with Dunnet's multiple comparison test. (^∗^ *p* < 0.05, ^∗∗^ *p* < 0.01, and ^∗∗∗^ *p* < 0.001).

The amount of mitochondrial DNA was determined by PCR 24 h and 72 h upon A*β*_1–42_ treatment or 18 h and 54 h after X-ray irradiation of SH-SY5Y cells cultivated at atmospheric oxygen (~21% O_2_) and at 5% O_2_, respectively. Effect of A*β* peptide or irradiation alone on mtDNA amount was dependent on O_2_ level in the cell culture: about 1.2- and 1.3-fold, respectively, decrease of mtDNA amount at 21% O_2_ and up to 1.3- and 1.5-fold, respectively, increase at 5% O_2_ compared to respective controls (Figures 8(a) and 8(b)). The combined effect of A*β* peptide and irradiation led to 1.8- and 1.4-fold, respectively, decrease in mtDNA amount at 21% and 5% O_2_, particularly at 21% O_2_ after 1 day or 18 h. However, no significant change in mtDNA amount at this oxygen concentration was observed 3 days after A*β* peptide treatment combined with irradiation, whereas at 5% O_2_ it was restored to the level of control sample. The presence of 142 bp PCR products in samples was a confirmation for a successful amplification (data in Figure S3).

### 3.7. Presence of Common mtDNA Deletion (Δ-mtDNA^4977^)

**Figure 9:**
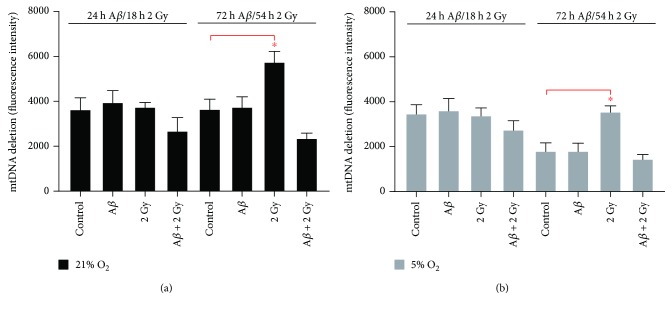
Common mtDNA deletion (Δ-mtDNA^4977^) upon A*β*_1–42_ treatment and/or irradiation in SH-SY5Y cells. (a, b) 2 Gy X-rays led to a significant increase in mtDNA deletion after 54 h at both 21% and 5% O_2_. Combination of A*β* peptide and irradiation resulted in less mtDNA deletion at both oxygen concentrations. Samples were measured in triplicates (*n* = 3) in four independent experiments (*N* = 4). Mean ± SEM analyzed by two-way ANOVA with Tukey's multiple comparison test. (^∗^ *p* < 0.05).

Common mitochondrial DNA deletion (Δ-mtDNA^4977^) was assayed using nested PCR for the detection of a very small amount of aberrant molecules in the sample [31]. The occurrence of the deletion can serve as an indicator of the increase in oxidative damage of mtDNA [25, 26], which may lead to changes in ATP concentration in the cell and results in cell death. Samples were amplified 24 h and 72 h upon A*β*_1–42_ treatment or 18 h and 54 h after irradiation (2 Gy X-rays) in SH-SY5Y cells cultivated at 21% and at 5% O_2_, respectively. No significant difference in Δ-mtDNA^4977^ was observed between control cells cultivated at 21% and at 5% O_2_. A significant increase (~1.6- and ~2-fold, resp.) in Δ-mtDNA^4977^ was observed only upon irradiation (2 Gy X-rays) after 54 h at both 21% and 5% O_2_ compared to corresponding controls (Figures 9(a) and 9(b)). A*β* peptide did not cause significant changes in mtDNA deletion measured at these two time points, whereas the combination of A*β* peptide and irradiation resulted in decreased Δ-mtDNA^4977^ at both oxygen concentrations (about 1.5- and 1.2-fold, resp.). The presence of the deletion was confirmed by agarose gel electrophoresis of PCR products in the form of 358 bp band (Figure S4).

### 3.8. Cell Death

**Figure 10:**
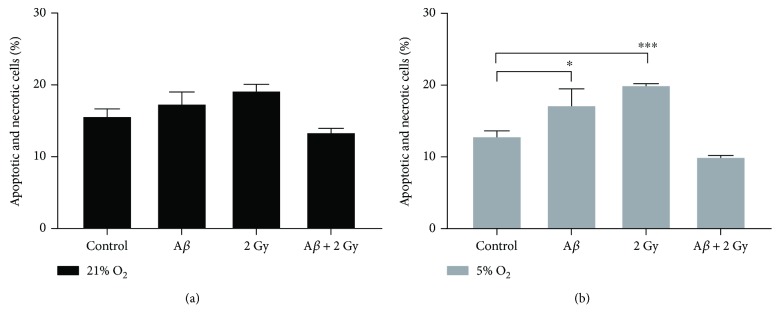
Percentage of apoptotic and necrotic cells after treatment with A*β*_1–42_ peptide and/or X-ray irradiation. (a) In cells cultivated at 21% O_2_, percentage of apoptotic and necrotic cells was only slightly increased (~1.1- or 1.2-fold) 24 h after A*β* peptide treatment or 18 h after irradiation (2 Gy), whereas combination of both resulted in a slight decrease in cell death (apoptosis and necrosis) compared to control cells. (b) In cells at 5% O_2_, A*β* peptide treatment or irradiation led to a significant (~1.3- or 1.5-fold) increase in percentage of dead cells. The combination of both amyloid beta treatment and irradiation resulted in the decrease (up to 1.3-fold) of cell death below its level in control sample. Samples were analyzed at least in duplicates (*n* = 2–6) in three independent experiments (*N* = 3), and data is analyzed by two-way ANOVA, followed by Tukey's posttests. (^∗^ *p* < 0.05 and ^∗∗∗^ *p* < 0.001).

Using Annexin V-FITC combined with propidium iodide staining, cells with loss of membrane integrity or cells with activated apoptosis were distinguished from healthy cells. Cells cultivated at 21% O_2_ showed an increase (~1.2-fold) in apoptosis and necrosis compared to cells at 5% O_2_. A*β* peptide or ionizing radiation led to an increase (~1.3- or 1.5-fold) in the number of early and late apoptotic as well as necrotic cells when cells were cultivated at 5% O_2_ (Figure 10(b)), whereas a nonsignificant increase (up to 1.2-fold for both stressors) in apoptotic and necrotic cells was observed at 21% O_2_ compared to control cells (Figure 10(a)). Interestingly, the combination of two stressors reduced cell death below the level present in control cells at both 21% and 5% O_2_.

## 4. Discussion

Effects of oxidative stress caused by ionizing radiation and/or by A*β*_1–42_ peptide on human neuroblastoma (SH-SY5Y) cells (pretreated with retinoic acid for induction of differentiation in order to obtain cells that resemble neurons) [37] were investigated. Since the level of oxidative stress depends on the oxygen concentration in the cell culture (Figures 4, 5, and 7), cells were cultivated in parallel at ~21% O_2,_ which is commonly used in cell culture incubators, and at more physiological 5% O_2_ that is close to the 4.6% O_2_ found in brain tissue [38]. We used TFA for disaggregation of the A*β*_1–42_ peptide, which effectively promotes the *β*-sheet (oligomeric) to random coil (monomeric) conversion of the peptide [29]. Our data revealed that externally applied disaggregated A*β*_1–42_ peptide (initially monomers and small oligomers with the size of ~4.5–30 kDa [39]) interacts with SH-SY5Y cells within minutes and the peak in the signal of fluorescently labelled peptide was reached after 18 h of incubation with cells (Figure 1). A slight decrease of the signal after 24 h suggests the peptide gets degraded or exported. In order to get information on subcellular localization of externally added A*β* peptide, we studied its colocalization with cellular organelles, that is, with endoplasmatic reticulum, mitochondria, and lysosomes. The peptide interacted preferentially with acidic organelles (lysosomes and possibly late endosomes) of SH-SY5Y cells cultivated at both 21% and 5% O_2_, respectively (Figure 2) and only at minor extent with other organelles (mitochondria and endoplasmatic reticulum) which showed only very weak colocalization with the peptide (Figure 3). It was previously demonstrated that cellular uptake of A*β*_1–42_ is independent of the attached fluorophore and nonattached fluorophore does not go into the cell [40]. However, the presence of the fluorophore might affect structural and functional properties of the peptide. Preparations of fluorescently labelled A*β* peptide should be compared with those of unlabelled peptide [41] in order to avoid atypical oligomers and fibrils. The confocal scanning microscopy used in this study proved A*β* peptide localization inside the cell, but due to the limited resolution, it cannot distinguish if the peptide only attaches to the membrane or if it penetrates in the membrane and enters cellular organelles. The peptide has a great potential to intercalate in all cellular membranes due to its hydrophobicity [13] and to cause structural perturbation, membrane fusion, changes in lipid diffusion, and dynamics [12, 42, 43]. Therefore, we expected its colocalization with the mitochondria or endoplasmatic reticulum as well. However, it was primarily found colocalized with lysosomes (Figure 2). These organelles present a powerful defense of the cell through its capability to digest unwanted compounds or damaged cellular structures. It seems that lysosomes represent very important defense against intracellular A*β* peptide toxicity [19]. Possibly, damaged parts of the mitochondria are packed in mitochondria-derived vesicles and are targeted to late endosomes and lysosomes for degradation and this process, although it shares similarities with bulk macrophagy and mitophagy, is specific for oxidative damage [44]. Although it is generally assumed that cytotoxic effects of A*β* peptides are exerted through the induction of ROS generation, similar to ionizing radiation, the observed increase in ROS level was not as high as expected (Figure 4). ROS generated after irradiation alone or combined with A*β* peptide was more pronounced in cells at 5% O_2_ as compared to atmospheric concentration. Control cells cultivated at 21% O_2_ had a much higher ROS level (Figure 4) and protein carbonylation (Figure 7) than cells at 5% O_2_. Probably, cultivation at nonphysiological 21% O_2_ already represents oxidative stress for cells. A variety of natural antioxidant mechanism that prevents ROS imbalance exists in the cell. For example, GSH is the primary defense mechanism against free radicals [34]. Control cells cultivated at 5% O_2_ had a higher GSH level than cells at 21% O_2_, and it was varying depending on the incubation duration with A*β* peptide and irradiation (Figure 5). Irradiation alone or in combination with A*β* peptide treatment had opposite effects depending on the oxygen concentration in the cell culture: significantly increased GSH level at 21% O_2_ and decreased at 5% O_2_, as compared to respective controls. Cultivation of cells at 21% O_2_ versus 5% O_2_ already presents a treatment, which can explain different levels of GSH after 24 h in controls in Figure 5(b) (nontreated cells cultivated at 21% and 5% O_2_, resp.). This difference was not present in controls after 6 h of incubation at different oxygen concentrations (Figure 5(a)) suggesting that longer time for adaptation of cells to 5% O_2_ is necessary. The other factor important in cellular repair processes is ATP. Its concentration was ~1.3-fold higher when cells were cultivated at 5% O_2_ as compared to 21% O_2_ (Figure 6). We observed a significant increase in ATP concentration after irradiation of A*β*-treated cells solely at 5% O_2_. The ATP concentration in the cell is balanced by its generation and consumption. The ATP concentration reflects the activity of the respiratory chain complexes and proliferation activity depending on irradiation and oxygen concentration. In addition, ATP is utilized for repair processes after oxidative stress or under other unfavorable conditions. Although cells activate their antioxidant defense mechanisms and repair pathways, intense or prolonged exposure to oxidative stress can disrupt or damage components of such pathways. Important targets of ROS in the cell, besides DNA, are proteins. Reactive electrophiles and other reactive oxygen species may induce irreversible alterations of protein structure and function leading to cellular dysfunction. For example, carbonyl groups are being introduced to proteins in reaction with ROS [45]. Carbonyl group (aldehyde or ketone) formation on protein side chains, mostly on prolines, arginines, lysines, and threonines, is a general biochemical marker of oxidative stress [36]. We observed generally a drastically higher (2.5-fold) level of protein carbonylation in cells cultivated at 21% O_2_ as compared to 5% O_2_ (Figure 7). A significantly pronounced further increase in protein carbonylation was detected only after irradiation of cells cultivated at 21% O_2_, whereas A*β* peptide alone or in combination with irradiation led only to a minor increase in protein carbonylation at both 21% and 5% O_2_ (Figure 7). This is in accordance with our assumption that protein damage at physiological oxygen concentration is less pronounced than at 21% O_2_. Also, protein repair and degradation systems are probably more efficient at 5% O_2_ compared to that at 21% O_2_. Since protein turnover is a dynamic process, damaged proteins are easily replaced by newly synthesized proteins as long as there are functional transcription and translation from nondamaged DNA. However, nucleic acids, particularly mtDNA, are very prone to oxidative damage by ROS causing mutations and larger rearrangements. We determined the relative amount of mtDNA and the occurrence of common mtDNA deletion (Δ-mtDNA^4977^) which occurs in diseases and aging after irradiation and is related to oxidative damage [25, 26]. It was suggested that the degeneration of neurons and synapses in AD may be associated with oxidative damage to nuclear DNA and more severe damage to mtDNA [46] leading to its degradation, a process unique to the mitochondrial compartment [47]. Although Δ-mtDNA^4977^ was proposed as one of the hallmarks in AD [48], another study correlated its presence with oxidative damage that occurs during aging but not with AD (not found in brains of AD patients) [49]. We observed a significantly increased level of the Δ-mtDNA^4977^ which was specific for irradiated cells only (Figure 9). The presence of the deletion 54 h after irradiation, and not 18 h after irradiation, is in accordance with our assumptions that effects of IR on mtDNA are rather delayed (accumulation of the damage by ROS) than direct. Also, the formation of deletions requires mtDNA replication (through the slip-replication mechanism) [25]. A*β* peptide treatment alone did not result in an increase in the amount of this deletion. Interestingly, in A*β*-treated cells which were subsequently irradiated, its amount was restored to the control level at both 21% and 5% O_2_. The amount of mtDNA detected by standard PCR was not significantly changed by A*β* peptide treatment alone or combined with irradiation at both 21% and 5% O_2_ (measured 72 h after treatment and 54 h after irradiation) (Figure 8). Noteworthy, irradiation of cells at 5% O_2_ resulted in a significant increase of mtDNA amount after 54 h (Figure 8). In general, cells at 5% O_2_ showed higher mtDNA amount after A*β* peptide treatment or after irradiation than cells at 21% O_2_. Probably, an increase in the mtDNA amount is a compensatory mechanism after stress and damage and it is dependent on the oxygen concentration in the cell culture. Although effects of irradiation and A*β* peptide share some similarities, such as induction of inflammation, these two stressors probably affect different cellular pathways and result in different cellular responses. Mitochondrial DNA deletions could be responsible for triggering compensatory mechanisms such as the increase in mtDNA amount (observed in cells at 5% O_2_ (Figure 8)) and improvement in mitochondrial respiration, which allows normal functioning of cells and may have a neuroprotective effect [50]. Also, balanced mitochondrial fission and fusion processes enable maintenance of their proper function and integrity including mtDNA quality [51] and it was reported that the mitochondria of cells cultivated at 5% O_2_ show larger mitochondrial networks and larger mitochondrial perimeters than those cultured at higher oxygen concentrations [52]. It was shown previously that A*β* peptide can induce oxidative-mediated autophagic cell death in vitro after damage of mtDNA in AD [53]. In addition, hyperoxic conditions (e.g., 95% O_2_) induce rapid cell death with fragmentation of mtDNA [54]. We analyzed induction of cell death (apoptosis and necrosis) in our system. A statistically significant increase in cell death was observed after A*β* peptide treatment or irradiation of cells at 5% O_2_, whereas this increase was only minor at 21% O_2_ (Figure 10). Surprisingly, the combination of both A*β* peptide and irradiation resulted in the decrease of cell death even below the level in control cells, particularly at 5% O_2_. Although GSH level decrease is used as an early indicator of apoptosis [55], in our study in most cases, we could not correlate it with cell death, particularly not in cells at 5% O_2_. Probably, cells employ other antioxidant and repair mechanisms depending on the starting level of oxidative stress. Unexpectedly, A*β* peptide did not harm SH-SY5Y cells to a great extent. The 4 *μ*M peptide applied in our studies probably represents a sublethal concentration for SH-SY5Y cell as reported previously for 10 *μ*M A*β* peptide applied to differentiated PC12 cells as well [56]. Additionally, cells were incubated with the peptide for 1 and 3 days, which are a short time compared to the many years that are necessary for action of the peptide in human brain. However, many nonlethal alterations in cell physiology such as a slight increase in protein carbonylation and interaction of A*β* peptide with lysosomes (in line with studies performed at 21% O_2_ on the same cell line [39]) if persistent may induce detrimental changes. Lysosomes, besides their digestive roles, have numerous other functions for the maintenance of cell integrity [57]. However, it is possible that continuous accumulation of the peptide harms the integrity and normal function of lysosomes. 4 *μ*M A*β* peptide did not result in a significant change in ROS level, GSH levels, mtDNA amount, and occurrence of mtDNA deletion (Figures 4, 5, 8, and 9). In some cases (Figure 10), the peptide even had protective functions, which requires further investigation. Possibly, aromatic amino acid residues of A*β* peptide act as ROS scavengers [35]. Furthermore, A*β* toxicity is cell-specific and depends on their metabolism; basal metabolism and its rate affect the level of endogenous oxidants and other mutagens [39, 58]. For example, picomolar concentrations of intracellular A*β*_1–42_ (both nonfibrillized and fibrillized) induce cell death of primary neurons through the p53-Bax pathway [59] but not of other neuronal and nonneuronal cell types. It was reported that some cell lines increased membrane fluidity and changed membrane properties and A*β* peptide processing in order to decrease the formation of toxic A*β* fragments [60]. Overexpression of mitochondrial transcription factor A (Tfam) is one of the mechanisms to compensate mitochondrial dysfunction, protect mtDNA from oxidative stress, and maintain mtDNA amount in SH-SY5Y cells treated with A*β* peptide [61].

## 5. Conclusions

The cellular response to stress is complex and depends on the cell type and its metabolism, starting level of oxidative stress, for example, oxygen concentration in the cell culture, differentiation state, and other factors.

Our study demonstrates that the oxygen concentration is an important factor that modulates cellular parameters and cell survival/death of neuronal-like human neuroblastoma cells in response to amyloid beta peptide treatment and X-ray irradiation.

Our data encourage the cultivation of cells at more physiological oxygen concentration for understanding radiation effects and disease mechanisms, particularly of complex disease such as AD, and developing potential therapeutics.

Lysosomes are an important target of A*β* peptide in the cell.

## Abbreviations

A*β*: Amyloid beta
AD: Alzheimer's disease
C-DCF: Carboxy-H_2_DCFH-DA (2′7′-dichlorofluorescin diacetate)
DMEM: Dulbecco's Modified Eagle's Medium
DPBS: Dulbecco's phosphate-buffered saline
FBS: Fetal bovine serum
FITC: Fluorescein isothiocyanate
RA: Retinoic acid
ROS: Reactive oxygen species
RT: Room temperature.

## Supplementary Materials

Supplementary Materials Figure S1: interaction of FITC-A*β* peptide with the mitochondria and endoplasmatic reticulum. Weak colocalization of FITC-A*β* peptide with the mitochondria stained by MitoTracker Red (red fluorescence) (A) and with endoplasmatic reticulum stained by ER-Tracker Red dye (red fluorescence) (B) at both 21% and 5% O_2_, documented after 3, 8, and 18 h. 400x magnification. 50 *μ*m scale bar. Figure S2: protein carbonylation. Oxyblot upon A*β*_1–42_ treatment and/or irradiation (2 Gy X-rays) in SH-SY5Y cells cultivated at 21% and 5% O_2_, respectively. Oxidized and derivatized (+) bovine serum albumin (BSA, 15 *μ*g, 60 kDa) served as a positive control and mass standard, and nonderivatized (−) BSA was used as a negative control for immunobinding. Mean gray values of Oxyblot lanes (~15 *μ*g protein/lane) were normalized to the gray value of BSA-positive control lane (mean grey value = 1). Figure S3: mitochondrial DNA amount upon A*β*_1–42_ treatment and/or irradiation in SH-SY5Y cells. The presence of 142 bp bands after PCR was evaluated by agarose gel electrophoresis on a 2% agarose gel with the addition of ethidium bromide for visualization of the DNA; 100 bp ladder was used as a molecular mass standard. Figure S4: common mtDNA deletion (Δ-mtDNA^4977^) upon A*β*_1–42_ treatment and/or irradiation in SH-SY5Y cells. The presence of a 358 bp band, which represents amplification of mtDNA harboring the deletion, was evaluated by agarose gel electrophoresis on 2% agarose gel with the addition of ethidium bromide; 100 bp ladder was used as a molecular mass standard.
