# Supplementary figures and images for: Oxygen Concentration and Oxidative Stress Modulate the Influence of Alzheimer's Disease Aβ 1–42 Peptide on Human Cells
Source: Oxid Med Cell Longev. 2018 Jan 11;2018:7567959. doi: 10.1155/2018/7567959 (PMC5821958; doi:10.1155/2018/7567959)

**Supplementary figures**

**Figure S1**
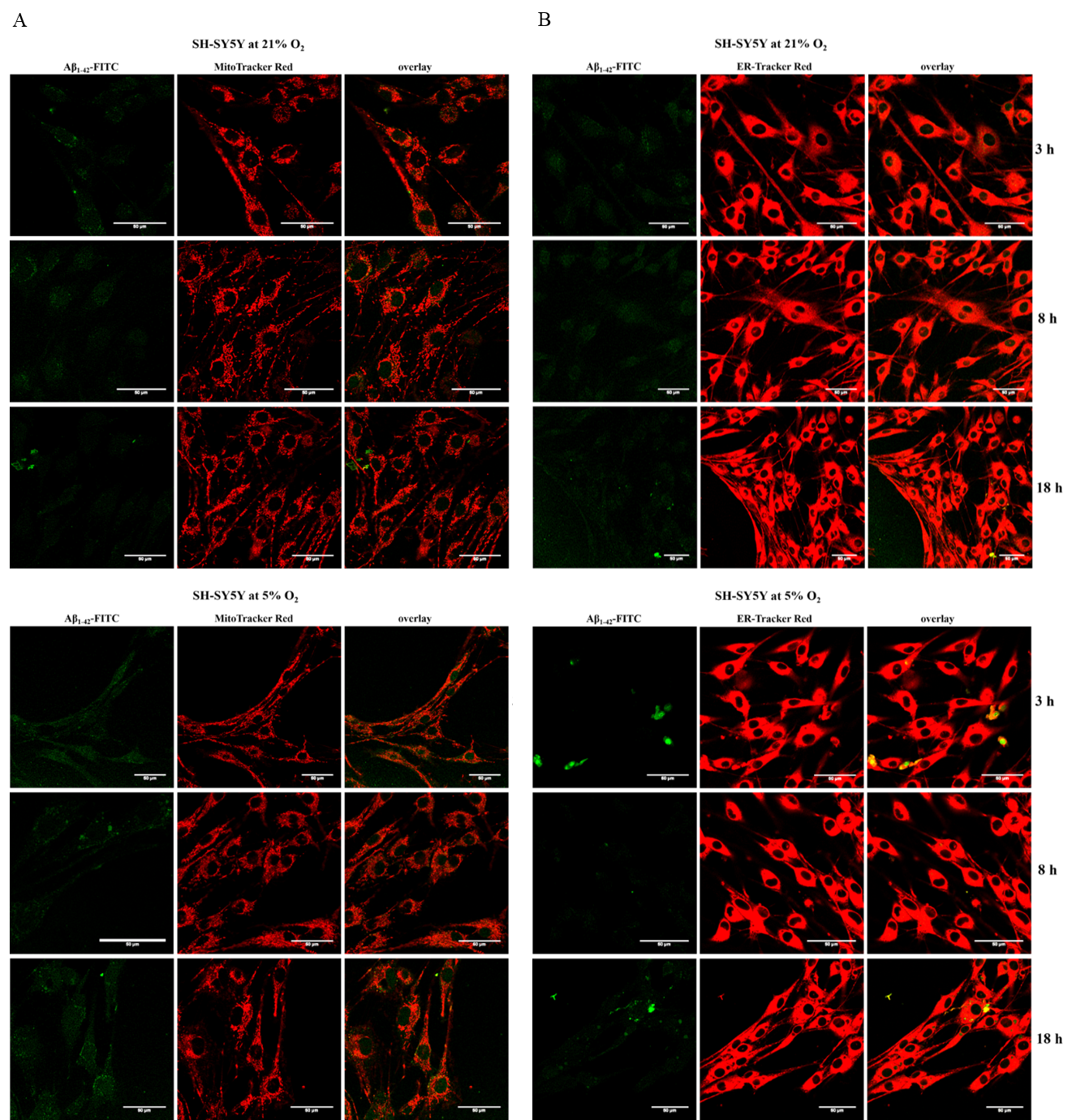


**Figure S2**


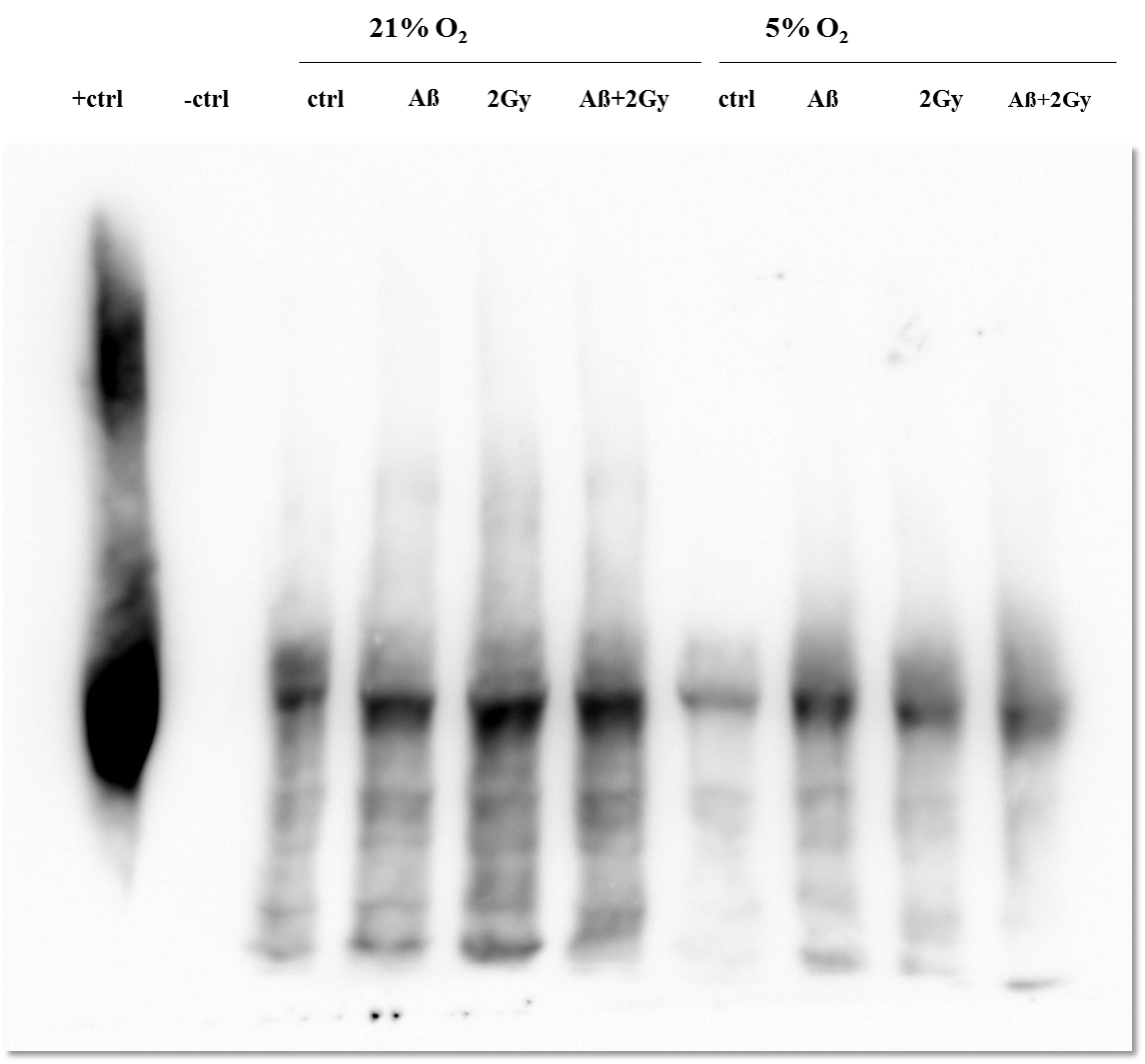


**Figure S3**





**Figure S4**

Supplement: Supplementary Materials — Figure S1: interaction of FITC-Aβ peptide with the mitochondria and endoplasmatic reticulum. Weak colocalization of FITC-Aβ peptide with the mitochondria stained by MitoTracker Red (red fluorescence) (A) and with endoplasmatic reticulum stained by ER-Tracker Red dye (red fluorescence) (B) at both 21% and 5% O2, documented after 3, 8, and 18 h. 400x magnification. 50 μm scale bar. Figure S2: protein carbonylation. Oxyblot upon Aβ 1–42 treatment and/or irradiation (2 Gy X-rays) in SH-SY5Y cells cultivated at 21% and 5% O2, respectively. Oxidized and derivatized (+) bovine serum albumin (BSA, 15 μg, 60 kDa) served as a positive control and mass standard, and nonderivatized (−) BSA was used as a negative control for immunobinding. Mean gray values of Oxyblot lanes (~15 μg protein/lane) were normalized to the gray value of BSA-positive control lane (mean grey value = 1). Figure S3: mitochondrial DNA amount upon Aβ 1–42 treatment and/or irradiation in SH-SY5Y cells. The presence of 142 bp bands after PCR was evaluated by agarose gel electrophoresis on a 2% agarose gel with the addition of ethidium bromide for visualization of the DNA; 100 bp ladder was used as a molecular mass standard. Figure S4: common mtDNA deletion (Δ-mtDNA4977) upon Aβ 1–42 treatment and/or irradiation in SH-SY5Y cells. The presence of a 358 bp band, which represents amplification of mtDNA harboring the deletion, was evaluated by agarose gel electrophoresis on 2% agarose gel with the addition of ethidium bromide; 100 bp ladder was used as a molecular mass standard. [file 7567959.f1.docx]
